# Adding Coronary Calcium Score to Exercise Treadmill Test: An Alternative to Refine Coronary Artery Disease Risk Stratification in Patients with Intermediate Risk Chest Pain

**DOI:** 10.5334/gh.766

**Published:** 2020-03-03

**Authors:** Weiting Huang, Zijuan Huang, Natalie Si Ya Koh, Jien Sze Ho, Terrance Siang Jin Chua, Swee Yaw Tan

**Affiliations:** 1 National Heart Centre Singapore, SG

**Keywords:** coronary artery calcium, exercise treadmill test, intermediate risk chest pain, risk stratification, obstructive coronary artery disease, health services research, computed tomography of coronary arteries

## Abstract

**Introduction:**

Chest pain is a common symptom for cardiology referrals. The ACC-AHA guidelines recommend exercise stress electrocardiography (TMX) as the initial diagnostic test. However, the TMX only has moderate sensitivity and non-diagnostic cases may require further stress imaging studies. In this study we aimed to look at the feasibility of combining coronary artery calcium (CAC) score with TMX to refine risk stratification. This may be an alternative to stress imaging in cases of non low-risk TMX, with the added advantage of short time turnaround time and low radiation dose.

**Methods:**

A total of 145 patients who presented consecutively to the National Heart Centre Singapore with chest pain were included in this study. These were intermediate risk patients with an average Duke Clinical Score of 38.8%. All patients underwent both TMX and computed tomography scan of the coronary arteries (CTCA) which also includes CAC. The primary outcome was defined as obstructive coronary artery disease i.e. >50% left main artery stenosis and/or >70% stenosis in other epicardial arteries.

**Results:**

The combination of TMX and CAC was comparable to stress imaging in detecting obstructive coronary artery disease. When added to TMX, CAC has an integrated discriminative improvement of 74.1%, achieved an area under the curve of 0.974 and adjusted R2 of 0.670 in detecting the primary outcome.

**Conclusion:**

The strategy of combining TMX and CAC is feasible in clinical practice to refine risk stratification in outpatients with intermediate risk chest pain. The calcium score readout also further guides therapy for primary prevention.

**Highlights:**

- Treadmill exercise is one of the first line tests for cardiac chest pain work up.
- Non diagnostic test treadmill results requires further adjunctive tests.
- Downstream stress imaging causes delay in time to diagnosis.
- Coronary artery calcium scoring needs minimal preparation with fast turnaround time.
- Addition of coronary artery calcium to treadmill performs comparably to stress imaging.
- This is a feasible alternative to risk stratify non diagnostic treadmill tests and guide therapy for primary prevention.

## Introduction

Chest pain is a common symptom for referral to the specialist cardiology clinic. The ACC-AHA guidelines on diagnosis and management of stable ischemic heart disease recommend exercise stress electrocardiography (TMX) as the initial diagnostic test, in patients at intermediate pretest risk who are able to exercise and have an interpretable resting electrocardiogram [1,2]. Advantages of an exercise treadmill test include lower cost, lower requirements for skilled technicians compared to stress imaging modalities, no radiation exposure and quick turnaround time.

However, the diagnostic accuracy of a TMX is only moderate. A meta-analysis of 24,047 patients in 147 studies found TMX to have sensitivity of 50% to 68% and specificity of 77% to 90% for detection of coronary artery disease [3]. Confounders such as resting ST-segment depression, digoxin usage, and left ventricular hypertrophy with repolarization changes decrease specificity, whereas mild single-vessel disease decreases sensitivity. An equivocal treadmill exercise test may incur more costs for downstream anatomical or stress imaging tests. Stress imaging modalities such as positron emission tomography (PET) and single-photon emission computed tomography (SPECT) have better sensitivities of approximately 80%. These tests help refine diagnostic accuracy but at cost of higher radiation exposure.

Due to the perceived costs and time for downstream testing, physicians may opt for more complex tests over TMX as the initial risk stratification strategy, which is non-ideal. In this paper we propose an alternative strategy of adding the computed tomography coronary artery calcium (CAC) score to TMX, to refine risk stratification and improve diagnostic accuracy. The combination of TMX with CAC score holds potential to better risk stratify patients, especially in cases of equivocal and non-low risk TMX.

Computed tomography coronary artery calcium (CAC) is a highly specific feature of coronary artery atherosclerosis. CAC is considered an appropriate test for asymptomatic patients at intermediate to high cardiovascular risk [4], but not in patients symptomatic with chest pain. There is a growing body of evidence showing that exercise capacity attenuates cardiovascular risk in asymptomatic patients with very high CAC score ≥ 400 and adds value to risk stratification [5,6]. Chang et al reported on the long-term prognostic findings when CAC score was added TMX testing in both symptomatic and asymptomatic low risk patients [7]. However, the diagnostic performance of the combination of TMX and CAC score for predicting significant coronary artery disease, especially on symptomatic chest pain patients, has not been tested.

In this study, we aim to evaluate the performance of the combination of TMX and CAC in diagnosing obstructive coronary artery disease.

## Methods

This was a cross sectional study. Recruitment occurred between 1^st^ January 2011 and 31^st^ December 2012. Patients referred to the National Heart Centre Singapore for evaluation of chest pain were approached to participate in this study. The National Heart Centre Singapore is a 185 bed national and regional referral centre for cardiovascular medicine. A total of 236 patients were approached to participate in our study. 91 patients declined participation. The sample size of our study cohort was 145 patients.

Baseline demographics, chest pain history, electrocardiogram and cardiovascular risk factors were collected at baseline by one clinical coordinator. The Duke Clinical Score (DCS) [8] was used to calculate the pre-test probability of significant obstructive coronary artery disease defined as left main artery stenosis ≥50% and/or any other epicardial artery stenosis of ≥70%.

All patients underwent 1) computed tomography coronary angiogram (CTCA) with readouts of degree of coronary artery stenosis and CAC score 2) TMX and 3) myocardial perfusion imaging (MPI) with tetrofosmin or thallium.

MPI Stress Testing: Patients underwent symptom limited exercise testing using a standard Bruce protocol or pharmacological stress testing. Exercise testing was conducted according to current guidelines, with tracer injection at peak stress, followed by maintenance of exercise for additional 1–2 minutes. Pharmacological stress-testing was performed with standardized protocols. Dipyridamole was infused at 0.142 mg·kg^–1^·minute^–1^ for 4 minutes, with aminophylline available for side-effects. For patients who were able to walk, the dipyridamole infusion was combined with low-intensity treadmill exercise. Dobutamine was infused using the standardized regimen of increasing rates from 5 to 40 μm·kg^–1^·minute^–1^, combined with atropine as necessary, until patients achieved 85% of their maximal predicted heart rate. Stress and rest imaging with either technetium (Tc-99 m) tetrofosmin or thallium was performed as per standard protocols [9]. Patients underwent stress imaging first, with rest imaging 4 h later or on a separate day. Imaging was performed following stress and during the resting state using dual-head gamma cameras (Philips CardioMD, Philips Vertex and ADAC Vertex) with step-and-shoot mode where acquisitions were performed over a 180° semicircular orbit with 64 stops. No attenuation correction was used.

All scans were reported with a 20-segment model, with segments graded using a 5-point scoring system ranging from 0 (normal) to 4 (absent perfusion) [10]. The score summed from the stress scan was defined as the summed stress score (SSS). As per standard practice, the difference between the summed stress and rest scores was termed the summed difference score (SDS), and reflected the amount of inducible ischemia present. Board-certified nuclear cardiologists reported all scans.

CTCA and CAC score procedures and reporting: The CTCA and CAC were performed using a Toshiba Aquilion ONE scanner with 160 mm coverage and 320 slice detector. Calcium scan was prospectively gated and scanned over a single heartbeat with a gantry rotation and x-ray exposure time of 0.35 second, 0.5-mm slice collimation, tube voltage of 120 kV, and tube current of 140 mA. Images were reconstructed at 3.0 mm slice thickness for calcium score. Assessment was carried out using Vitrea Calcium software and Agatston scoring schema. All studies were assessed for arterial lumen stenosis for all coronary arterial segments. Images were assessed using volume-rendered images, curved multiplanar reformations, and cross-sectional images in available phases as well as from sharp and standard kernels. Visual assessment of arterial segment lumen diameter stenosis was carried out. In assessing stenosis, the minimum lumen diameter was identified for each arterial segment and then compared with a reference site of a disease-free site in closest proximity to the lesion site. Disagreements between the two readers was resolved by consensus. Primary outcome of significant obstructive coronary artery disease is defined left main artery stenosis ≥50% and/or any other epicardial artery stenosis of ≥70% on CTCA [11]. Determination of outcome of significant coronary artery stenosis on CTCA was by 2 independent radiologists who were not involved in the baseline data collection.

The study complied with the Declaration of Helsinki and was approved by our Centre’s Institutional Review Board.

## Statistical Methods

Continuous normally distributed variables are compared by t-test and categorical variables by chi square test in univariate analysis.

We built 3 logistic regression models, comparing the Duke clinical score with Duke Treadmill Score (Model 1), Duke clinical score with Duke Treadmill Score and CAC (Model 2) and Duke clinical score, Duke Treadmill score with myocardial perfusion imaging (Model 3). Discriminative ability of the different models was evaluated by the area under to receiver operating curve and intergrated discrimination improvement (IDI) [12], and goodness-of-fit by the Hosmer-Lemeshow test.

Internal validation was further tested by K-fold cross validation. Category-free net reclassification improvement (NRI) [12] was also assessed. For ease of practical interpretation in clinic, we assessed the sensitivity, specificity, positive and negative predictive values using guideline advocated cut-offs of calcium score 100 and 400 [13] in patients with low and non-low risk duke treadmill scores.

All statistical analysis were performed on Stata Version 14.0 (StataCorp. 2015. College Station, TX: StataCorp LP). A p-value of <0.05 was considered as statistically significant.

## Results

A total of 145 patients were recruited for this study. All patients were in the intermediate risk category as classified by the Duke clinical score. Baseline demographics are presented in Table 1. A total of 23 patients had significant epicardial coronary artery stenosis on CT coronary angiogram.

**Table 1:** Baseline Demographic Table of Study Participants.

Clinical Variables	Total Population (n = 130)	Patients with significant stenosis on CTCA (n = 23)	p-value*

**Age (years)**	55.79 ± 12.37	58 ± 9.54	0.347
**Male gender**	89 (61.38%)	17 (73.91%)	0.184
**Diabetes Mellitus**	37 (25.69%)	10 (43.48%)	0.065
**Hypertension**	89 (59.72%)	15 (65.22%)	0.531
**Hyperlipidemia**	107 (73.79%)	20 (86.96%)	0.285
**Family History of premature CAD**	60 (41.38%)	12 (52.17%)	0.255
**Current Smoker**	14 (9.66%)	5 (21.74%)	0.041
**Chest Pain**			
***Typical Angina***	18 (12.41%)	6 (26.09%)	0.077
***Atypical Angina***	88 (60.69%)	12 (52.17%)	0.901
***Non cardiac chest pain***	39 (26.90%)	5 (21.74%)	Ref
**Duke Clinical Score (%)**	38.8 ± 13.7%	31.7 ± 8.2%	0.009
**Duke Treadmill Score**			
***Low risk***	79 (67.52%)	4 (21.05%)	Ref
***Intermediate risk***	35 (29.91%)	12 (63.16%)	0.001
***High risk***	3 (2.56%)	3 (15.79%)	0.003
**CT Calcium Score**			
**0**	37 (25.52%)	0	Ref
**1–99**	51 (35.17%)	2 (8.70%)	0.395
**100–400**	32 (22.07%)	4 (17.39%)	0.102
**>400**	25 (17.24%)	17 (73.91%)	0.001
**Moderate to severe ischemia on MPI**	15 (10.34%)	11 (47.83%)	0.001
**Cardiovascular Event and All Cause Death**	17 (11.72%)	11 (47.83%)	0.001

* p-value compares the groups with and without significant stenosis on CTCA.

**Diagram 1:**
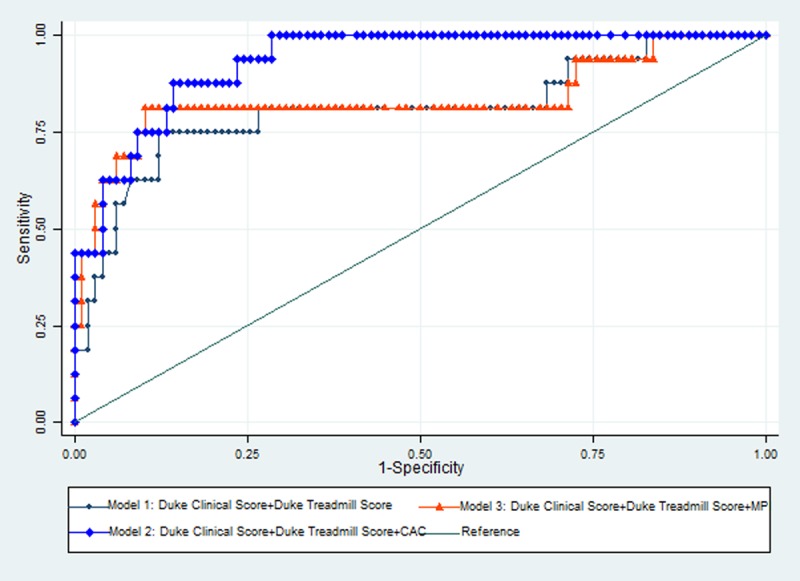
ROC curve of Model 1, Model 2 and Model 3.

The Duke clinical score, duke treadmill score, CAC score and a positive myocardial perfusion scan were univariately significantly associated with the diagnosis of >70% epicardial artery stenosis on CTCA. We build logistic regression models to predict the primary outcome: Model 1 (Duke Clinical Score and Duke treadmill Score), Model 2 (Duke Clinical Score, Duke Treadmill Score and CAC) and Model 3 (Duke Clinical Score, Duke Treadmill Score and Myocardial Perfusion Imaging). The comparisons of coefficients of Models 1, 2 and 3 are presented in Table 2. Comparing model performance using ROC curves, Model 2 (Duke Clinical Score, Duke Treadmill Score and CAC) performed significantly better than Model 1 (Duke Clinical Score and Duke treadmill Score); p-value of 0.016. Model 2 also performed comparably to Model 3(Duke Clinical Score, Duke Treadmill Score and Myocardial Perfusion Imaging) with a non significant p-value of 0.086 (see Diagram 1 for ROC curve comparisons).

**Table 2:** Univariate and Multivariate Logistic Regressions for Outcome of Epicardial artery stenosis >70% on CTCA.

Variables	Univarate		Model 1 (Duke Clinical score + TMX)		Model 2 (Duke Clinical score + TMX + CAC)		Model 3 (Duke Clinical Score + TMX + MPI)	

	Odds Ratio (SE)	p-value	Odds Ratio (SE)	p-value	Odds Ratio (SE)	p-value	Odds Ratio (SE)	p-value

Duke Clinical Score	0.28 (0.13)	0.010	1.31 (1.61)	0.017	1.61 (2.41)	0.211	1.44 (1.82)	0.055
Duke Treadmill Score								
Low Riskⱡ	1.01.0		1.0		1.0		1.0	
Intermediate Risk	5.94 (1.62)	0.001	7.11 (1.77)	0.001	9.68 (3.25)	0.003	6.80 (1.94)	0.001
High Risk	12.85 (4.31) 3.52 (1.26)9	0.003	12.76 (4.32)	0.003003	15.27 (5.36)	0.0040.	11.67 (4.75)	0.0140
Coronary Calcium Score (per unit)	0.018 (0.003)	0.001			0.018 (0.004)	0.001		
MPI ischemia							7.57 (2.59)	0.004
None or mild ischemiaⱡ	1.0							
Moderate to Severe	8.66 (1.71)	0.001						
Ischemia								
AUC C-statistic			0.833 (0.710–0.956)		0.974 (0.947–1.000)		0.833 (0.686–0.980)	
Adjusted R2			0.257		0.670		0.336	

The goodness-of-fit of the new combination of Duke clinical score, Duke treadmill score and CAC score was demonstrated by a non-significant Hosmer-Lemeshow test of 0.917. Internal validation of the model was also evaluated by k-fold validation.

We also explored the Net reclassification improvement and IDI after adding CAC score to the Duke Clinical Score and TMX (Model 2 versus Model 1). The IDI was 16.8% with a significant p-value of 0.001. The category-free event NRI was 0.47 (95%CI 0.07–0.87) and non-event NRI was 0.86 (95%CI 0.73–0.96).

For ease of interpretation at a clinic setting, we classified patients into 2 groups of low risk and non-low risk treadmill scores. Using previously defined coronary artery calcium cut-offs of 100 and 400 for association with moderate non-obstructive coronary artery disease and significant coronary artery stenosis respectively, we presented a contingency table with readouts of sensitivity, specificity, positive and negative predictive value for patients in these 2 risk groups (please see Table 3).

**Table 3:** Interpretation of Duke Treadmill Score with Calcium Score.

			Sensitivity	Specificity	Positive Predictive Value	Negative Predictive Value

**Low risk Duke Treadmill Score**						
	*CAC* < *100*	*CAC* ≥ *100*				
*Presence of significant CAD*	0	4	100%	77.3%	19.0%	100%
*Absence of significant CAD*	58	17				
	*CAC* < *400*	*CAC* ≥ *400*				
*Presence of significant CAD*	0	4	100%	96%	57.1%	100.0%
*Absence of significant CAD*	72	3				
**Intermediate to High risk Duke Treadmill Score**						
	*CAC* < *100*	*CAC* ≥ *100*				
*Presence of significant CAD*	2	13	86.7%	65.2%	61.9%	88.2%
*Absence of significant CAD*	15	8				
	*CAC* < *400*	*CAC* ≥ *400*				
*Presence of significant CAD*	6	9	60.0%	91.3%	81.8%	77.8%
*Absence of significant CAD*	21	2				

## Discussion

CAC score adds value, both as a combination test with TMX and potentially sequential test to refine risk stratification in non-low risk or equivocal TMX. It is a rapid test that can be performed on any CT scanner with low radiation exposure at ~1 mSV, as compared to nuclear stress tests (~10 to 22mSV) and CTCA (3 to 5 mSV). It is also relatively inexpensive and does not require iodinated contrast agents. However, the CAC score has a low specificity for predicting major adverse cardiovascular events, especially if the CAC value of >0 is used (35%). Specificity increased with higher cut off values (>100, 67%; >400, 85%) but at the expense of sensitivity (61% and 31%, respectively) [14]. Stress testing detects the functional consequences of atherosclerosis but does not diagnose atherosclerosis because myocardial ischemia can be caused by other conditions such as microvascular disease or left ventricular hypertrophy. The combination of the TMX and the CAC score attempts to improve diagnostic accuracy by combining pathological changes in both coronary anatomy and myocardium with development of fixed coronary artery stenosis in the setting of angina.

We chose to use the CAC score as a continuous variable as we believe that although cut-offs as mentioned above help in prognostication of MACE, a continuous scale adjusted by the Duke clinical score, which takes into the factor of age, may improve sensitivity of diagnosis of obstructive coronary artery disease.

The combination test of TMX and CAC score may change the way the health services are arranged. The TMX is often supervised by a trained technician or physician who usually can give an interpretation of the TMX immediately after the test. An equivocal or non-low risk test, especially intermediate risk test, may be scheduled to proceed with a CAC score which does not require any pre-procedure preparation and has a quick turn-around time. This will save patient and physician an additional visit to review an abnormal test and discussion for another stress imaging test. It can also refine risk stratification in non-low risk patients on TMX prior to coronary angiography.

Additionally, the calcium score also guides therapy for primary prevention. In groups of patients with atherosclerotic cardiovascular disease 10-year risk of 5% or more, which are represented by the patients in this cohort, CAC > 0 should prompt initiation of statin therapy for primary prevention [15]. Such practice can have greater impact in reducing future myocardial infarction risk, compared to discharging patients with a normal treadmill without a discussion of future cardiovascular risk and modulation through statin therapies.

This study is a prospective trial design, but the small sample size affects the power of the study. Nevertheless our analysis shows that the combination of the Duke clinical score, TMX and CAC score is superior than Duke clinical score and TMX alone and, and probably even current stress imaging techniques of myocardial perfusion imaging in diagnosing obstructive coronary artery disease.

There is a follow-up ongoing larger study to externally validate our findings as above.

## Conclusion

The addition of CAC score improves diagnosis of obstructive coronary artery disease compared to Duke clinical score and TMX alone. It has potential to be a rapid test to refine risk stratification in non-low risk or non-diagnostic TMX tests.
